# The impact of COVID‐19 on multi‐month dispensing (MMD) policies for antiretroviral therapy (ART) and MMD uptake in 21 PEPFAR‐supported countries: a multi‐country analysis

**DOI:** 10.1002/jia2.25794

**Published:** 2021-10-28

**Authors:** Lauren E. Bailey, George K. Siberry, Patricia Agaba, Meaghan Douglas, Jessica R. Clinkscales, Catherine Godfrey

**Affiliations:** ^1^ United States Agency for International Development Office of HIV/AIDS Washington DC USA; ^2^ U.S. Military HIV Research Program Walter Reed Army Institute of Research Silver Spring Maryland USA; ^3^ Henry M. Jackson Foundation for the Advancement of Military Medicine Bethesda Maryland USA; ^4^ U.S. Department of State Office of the Global AIDS Coordinator Washington DC USA

**Keywords:** ART, COVID‐19, MMD, PEPFAR, treatment continuity, viral load

## Abstract

**Introduction:**

Increasing access to multi‐month dispensing (MMD) of antiretroviral therapy (ART) supports treatment continuity and viral load suppression for people living with HIV (PLHIV) and reduces burden on health facilities. During the COVID‐19 response, PEPFAR worked with ministries of health to scale up MMD and expand eligibility to new groups of PLHIV, including children and pregnant/breastfeeding women. We analysed PEPFAR program data to understand the impact of the policy changes on actual practice.

**Methods:**

We conducted a desk review in 21 PEPFAR‐supported countries to identify and collect official documentation released between March and June 2020 addressing changes to MMD guidance during the COVID‐19 response. MMD coverage, the proportion of all ART clients on MMD, was assessed in the calendar quarters preceding the COVID‐19 response (Q4 2019, October–December 2019; and Q1, January–March 2020) and the quarters following the start of the response (Q2 2020, April–June 2020; Q3 2020, July–September, 2020; Q4 2020, October–December 2020). We used the two‐proportion Z‐test to test for differences in MMD coverage pre‐COVID‐19 (Q4 2019) and during implementation of COVID‐19 policy adaptations (Q2 2020).

**Results and discussion:**

As of June 2020, 16 of the 21 PEPFAR‐supported countries analysed adapted MMD policy or promoted intensified scale‐up of MMD in response to COVID‐19. MMD coverage for all clients on ART grew from 49% in Q4 2019 pre‐COVID‐19 to 72% in Q2 2020 during COVID‐19; among paediatric clients (< 15), MMD coverage increased from 27% to 51% in the same period. Adaptations to MMD policy were associated with a significantly accelerated growth in the proportion of clients on MMD (*p* < 0.001) for all populations, irrespective of age and dispensing interval.

**Conclusions:**

Access to MMD markedly expanded during the COVID‐19 pandemic, supporting treatment continuity while mitigating exposure to COVID‐19 at health facilities. This model is beneficial in public health emergencies and during disruptions to the healthcare system. Outside emergency contexts, expanded MMD eligibility extends client‐centred care to previously excluded populations. The success in expanding MMD access during COVID‐19 should motivate countries to recommend broader MMD access as a new standard of care.

## 1 INTRODUCTION

Policies permitting multi‐month dispensing (MMD) of antiretroviral therapy (ART) have become increasingly common, allowing people living with HIV (PLHIV) to reduce the frequency of ART pickups. In 2016, the World Health Organization (WHO) recommended less frequent medication pickup (3‐ to 6‐month intervals) for clients “stable on ART” and reinforced the provision of MMD, particularly 6‐month multi‐month dispensing (6MMD), for clients responding well to treatment in the updated 2021 guidelines [1, 2]. MMD has an important impact on individual treatment success and there is a growing body of literature showing improved treatment continuity and viral load suppression for people in MMD models [3, 4, 5]. Extended ART dispensing intervals improve client satisfaction and ease the burden on stretched health facilities by reducing health worker workloads and decongesting clinics [6, 7, 8, 9]. MMD, specifically 6MMD, is also associated with cost savings to both the healthcare system and patients [10].

The United States President's Emergency Plan for AIDS Relief (PEPFAR) encourages MMD as a key strategy for promoting client‐centred differentiated care and treatment continuity [11]. The scope of MMD policies, eligibility criteria and implementation varied greatly across PEPFAR‐supported countries prior to the COVID‐19 pandemic; however, MMD uptake was increasing globally and PEPFAR was promoting MMD as a minimum program requirement per its 2019 Country Operational Plan guidance [12]. An internal 2020 PEPFAR MMD policy analysis, which reviewed MMD policies in national HIV treatment guidelines in 21 PEPFAR‐supported countries, revealed that most countries permitting MMD limited it to adults who were “stable on ART”, though the definition of “stable on ART” differed slightly across countries and did not always align with the 2016 WHO definition, which requires: receiving ART for at least 1 year, no current illnesses or pregnancy, a good understanding of lifelong adherence and evidence of treatment success [1].

In March 2020, in anticipation of COVID‐19‐related disruptions in supply chain, facility operations and patient mobility, PEPFAR released technical guidance highlighting reinforcement and expansion of MMD as a means to mitigate potential exposure to COVID‐19 at health facilities and protect HIV treatment continuity during the pandemic [13]. Also beginning in March 2020, several PEPFAR‐supported countries began disseminating updated MMD guidelines for expanded access to MMD. PEPFAR worked with ministries of health to institute and implement these guidelines during the COVID‐19 response.

PEPFAR used an iterative process to ensure a sufficient supply of antiretrovirals (ARVs) to scale MMD, including: analysing stock levels using the electronic Logistics Management Information System (eLMIS), placing early orders, developing and implementing ARV distribution plans and conducting regular follow‐up with site pharmacists. PEPFAR also supported health worker trainings on the updated MMD guidelines, conducted supportive supervision visits to health facilities and utilized MMD focal persons to review client registers and identify clients eligible for MMD.

We analysed PEPFAR program data to understand the impact of the policy changes on actual practice and explored the potential benefits of adopting COVID‐19 adaptations to MMD policy and broader MMD access as the standard of care.

## 2 METHODS

### 2.1 COVID‐19 MMD policy analysis

We conducted a desk review with support from PEPFAR colleagues at overseas United States (US) government missions to identify and collect official documentation released between March and June 2020 addressing MMD guidance during the COVID‐19 response. We reviewed the documentation against pre‐COVID guidelines noting any changes to the policy. Policy adaptations were: longer dispensing intervals; expanded eligibility criteria, including changes to minimum time on ART, age requirements, viral load status, treatment regimen type, pregnancy and breastfeeding status, and “stable on ART” status; and intensification or promotion of MMD without actual policy change.

### 2.2 MMD data collection and analysis

PEPFAR programs routinely collect quarterly MMD data from PEPFAR‐supported ART sites in over 20 countries and three regional programs using ministry of health clinical data collection tools, electronic medical records or program monitoring tools and report the data in PEPFAR's electronic data reporting system. Due to country‐specific reporting limitations, South Africa, Ukraine, Botswana and Namibia were excluded, leaving 21 PEPFAR‐supported country programs included in this analysis. The Asia, West Africa and western Hemisphere Regional PEPFAR programs were also excluded due to the disproportionately small size of the programs and incomplete data.

All PEPFAR‐supported clients on treatment are categorised as receiving one of three ARV‐dispensing frequencies: less than 3 months, 3–5 months (3–5MMD) and 6 or more months. PEPFAR defines MMD as receiving at least 3 months of ARVs and disaggregates MMD data by dispensing interval (3–5MMD and 6MMD) and by sex and coarse age disaggregates (15+ and <15 years). We looked at MMD coverage, the proportion of all ART clients on MMD, receiving MMD in the quarters directly preceding the global COVID‐19 response (Q4 2019, October–December 2019; and Q1 2020, January–March 2020) and the immediate quarters following the start of the global COVID‐19 response (Q2 2020, April–June 2020; Q3 2020, July–September; and Q4 2020, October–December 2020).

We also performed a two‐proportion *z* test in R to test for differences in MMD coverage pre‐COVID in Q4 2019 and during implementation of COVID‐19 adaptations in Q2 2020. We compared Q4 2019 rather than Q1 2020 to Q2 2020 in the *z* test as some COVID‐19 adaptations were already being implemented in March at the end of Q1 2020.

### 2.3 Viral load data collection

PEPFAR also collects quarterly viral load testing data from laboratory or medical records for all clients on ART. PEPFAR calculates viral load suppression as the proportion of documented viral load results from adult and paediatric ART patients who have been on ART for at least 3 months with a viral load result of <1000 copies/ml.

### 2.4 Ethical approval

This was an analysis of facility‐level aggregated program data and did not require Institutional Review Board approval or consent.

## 3 RESULTS AND DISCUSSION

### 3.1 COVID‐19 adaptations to MMD policy

We identified documentation of MMD policy adaptations or directives to scale‐up MMD for 16 of the 21 PEPFAR‐supported countries in the analysis. Specific policy adaptations varied with some countries recommending MMD for nearly all ART clients, while other countries enacted narrower policy adaptations that expanded eligibility for specific sub‐populations or increased dispensing intervals (Table 1).

**Table 1:** Country‐specific COVID‐19 adaptations to MMD policy [14, 15, 16, 17, 18, 19, 20, 21, 22, 23, 24, 25, 26, 27, 28, 29, 30, 31, 32]

		COVID‐19 adaptation category	
Country	Pre‐COVID‐19 MMD policy/practice	Increased dispensing intervals	Expanded eligibility details
Burundi	3MMD for clinically stable^a^, ^c^	No	3MMD for clinically stable and unstable clients, children and PBFW on first‐line regimen; 2MMD for clients on second‐ or third‐line regimen
Cote d'Ivoire	3‐6MMD for clinically stable^b^, ^c^	No	3MMD for new ART initiators and clinically unstable clients
Democratic Republic of the Congo (DRC)	3MMD for clinically stable^a^	6MMD	3MMD for new clients who have been on ART for 3 months; 6MMD for clients on ART for > 3 months
Dominican Republic	No policy	6MMD	6MMD for clinically stable clients; 3MMD for clinically unstable clients
Eswatini	3MMD for clinically stable^a^, ^c^	6MMD	3MMD for virally suppressed children > 2 years; 3MMD for all clients on first‐line TLD; 3MMD for stable, virally suppressed clients on second‐line DTG‐based regimens; 6MMD for all clients on first‐line TLE; 3MMD for eligible, new ART initiators
Ethiopia	3‐6MMD for clinically stable^b^	No	3MMD for PBFW, paediatrics, new ART initiators, clients on second‐ and third‐line ART and clinically unstable clients not needing readmission
Kenya	3MMD for clinically stable^a^, ^c^	No	Up to 3MMD for all PLHIV regardless of age and viral load status (does not include PBFW and new‐ART initiators)
Lesotho	3MMD for clinically stable^a^, ^c^	6MMD	3–6MMD for all eligible clients including stable children > 2 years, adolescents and PBFW
Malawi	3‐6MMD for clinically stable^a^	6MMD	6MMD for clients > 20 kg, new ART initiators (on ART for 3 months) and suppressed VL in the last 6 months is not required; 3MMD for PBFW
Mozambique	3MMD for clinically stable^a^, ^c^	No	3MMD for new ART initiators (on ART for 3 months), children > 2 years and PBFW
Uganda	3MMD for clinically stable^a^, ^d^	No	No age limits for 3MMD (this does not include clients on second‐ or third‐ line ART, new ART initiators, virally non‐suppressed clients, lactating mothers with babies < 6 months and the very sick)
Zambia	3‐6MMD for clinically stable^a^, ^c^	6MMD	3MMD for children 2–10 years; 6MMD for adolescents 10–19 years; 3–6MMD for clients with comorbid conditions; 3MMD for clients failing treatment and receiving enhanced adherence counselling; All health facilities providing ART must ensure recipients of care in contact with the facility receive 6MMD
Zimbabwe	3MMD for clinically stable^a^	6MMD	6MMD for priority groups: PLHIV > 50 years, clients with comorbidities and adolescents
Haiti	3‐6MMD for clinically stable^a^, ^d^	No policy change, but guidance issued to intensify scale‐up of MMD	
South Sudan	3‐6MMD for clinically stable^a^	No policy change, but guidance issued to intensify scale‐up of MMD	
Tanzania	3‐6MMD for clinically stable^a^	No policy change, but guidance issued to intensify scale‐up of MMD	
Angola	3MMD for clinically stable^a^, ^c^	Unknown/official documentation not located	
Cameroon	3‐6MMD for clinically stable^a^	Unknown/official documentation not located	
Nigeria	3‐6MMD for clinically stable^a^, ^c^	Unknown/official documentation not located	
Rwanda	3MMD for clinically stable^a^, ^d^	Unknown/official documentation not located	
Vietnam	3MMD for clinically stable^c^	Unknown/official documentation not located	

^a^ Minimum age and/or weight requirements.

^b^ Pregnant and/or breastfeeding women not included.

^c^ Age requirements not specified.

^d^ First‐ or second‐line ART only.

ART, antiretroviral therapy; DTG, dolutegravir; MMD, multi‐month dispensing; PBFW, pregnant/breastfeeding women; PLHIV, people living with HIV; TLD, tenofovir lamivudine dolutegravir; TLE, tenofovir lamivudine efavirenz; VL, viral load; 3MMD, 3‐month multi‐month dispensing; 6MMD, 6‐month multi‐month dispensing.

### 3.2 Changes in MMD coverage

MMD policy adaptations implemented during the COVID‐19 response (Q2 2020) were associated with a significantly accelerated growth in MMD coverage (*p* < 0.001), irrespective of age or dispensing interval. MMD coverage for all clients on ART grew substantially from 49% (5,093,561/10,372,711) in Q4 2019 pre‐COVID‐19 to 72% (8,051,775/11,121,591) in Q2 2020 during COVID‐19; and among paediatric clients, MMD coverage grew from 27% (142,580/524,546) in Q4 2019 to 51% (270,984/531,538) in Q2 2020. Across MMD dispensing intervals, 3–5MMD for all clients on ART increased from 40% (4,180,036/10,372,711) in Q4 2019 to 55% (6,134,728/11,121,591) in Q2 2020; and among paediatric clients, 3–5MMD increased from 24% (127,261/524,546) to 45% (238,561/531,538) in the same period. The proportion of clients on ART receiving 6MMD increased from 9% (913,525/10,372,711) in Q4 2019 to 17% (1,917,047/11,121,591) in Q2 2020; and among paediatric clients, 6MMD coverage doubled from 3% (15,319/524,546) to 6% (32,423/531,538) in the same period (Tables 2 and 3).

**Table 2:** Proportion and absolute number of all ART clients on MMD in 21 PEPFAR‐supported countries (October 2019–December 2020)

Quarter	Clients on ART	3–5MMD (%)	6MMD (%)	Total MMD (%)
Q4 2019	10,372,711	4,180,036 (40%)	913,525 (9%)	5,093,561 (49%)
Q1 2020	10,703,679	5,198,528 (49%)	1,014,704 (9%)	6,213,232 (58%)
Q2 2020^1^	11,121,591	6,134,728 (55%)^a^	1,917,047 (17%)^b^	8,051,775 (72%)^c^
Q3 2020	11,476,916	6,196,129 (54%)	2,308,130 (20%)	8,504,259 (74%)
Q4 2020	11,656,878	6,227,107 (53%)	2,517,943 (22%)	8,745,050 (75%)

^1^Two‐proportion Z‐test comparing MMD % for Q2 2020 (COVID‐19) versus Q4 2019 (pre‐COVID‐19): (a) for 3–5 MMD, *p*<0.001; (b) for 6MMD, *p* < 0.001; and (c) for total MMD, *p* <0.001.

ART, antiretroviral therapy; MMD, multi‐month dispensing; PEPFAR, the United States President's Emergency Plan for AIDS Relief; 3‐5MMD, 3‐5‐month multi‐months dispensing; 6MMD, 6‐months multi‐month dispensing.

**Table 3:** Proportion and absolute number of paediatric (<15 years) ART clients on MMD in 21 PEPFAR‐supported countries (October 2019–December 2020)

Quarter	Paediatric clients on ART	3–5MMD (%)	6MMD (%)	Total MMD (%)
Q4 2019	524,546	127,261 (24%)	15,319 (3%)	142,580 (27%)
Q1 2020	525,128	165,620 (32%)	16,055 (3%)	181,675 (35%)
Q2 2020^1^	531,538	238,561^a^ (45%)	32,423^b^ (6%)	270,984^c^ (51%)
Q3 2020	537,126	242,968 (45%)	38,792 (7%)	281,760 (52%)
Q4 2020	532,239	244,351 (46%)	39,814 (7%)	284,165 (53%)

^1^Two‐proportion Z‐test comparing MMD % for Q2 2020 (COVID‐19) versus Q4 2019 (pre‐COVID‐19): (a) for 3–5 MMD, *p* < 0.001; (b) for 6MMD, *p* <0.001; and (c) for total MMD, *p* < 0.001.

MMD growth slowed considerably following the Q2 2020 surge, likely due to select countries achieving saturation of MMD enrolment among eligible clients or disruptions to the ARV supply chain due to COVID‐19, but total MMD coverage continues to grow across the PEPFAR program. There is a drop‐off in the proportion of clients receiving 3–5MMD starting in Q3 2020 and continuing in Q4 2020 and a subsequent increase in the proportion of clients receiving 6MMD as programs begin transitioning more clients from 3–5MMD to 6MMD.

### 3.3 Changes in MMD coverage in select countries

Notable increases in MMD coverage among PEPFAR‐supported clients were observed in a number of countries during the COVID‐19 response; though tests of statistical significance were not performed on individual countries. In Ethiopia and the Democratic Republic of the Congo (DRC), where the governments recommended MMD for nearly all ART clients starting in March and April 2020, respectively; total MMD coverage in Ethiopia was 41% in Q4 2019, climbed to 79% in Q2 2020, and reached 89% in Q4 2020; and in DRC, total MMD coverage was 35% in Q4 2019, increased to 88% in Q2 2020 and reached 94% in Q4 2020. Among clients < 15 years of age, total MMD coverage in Ethiopia increased from 14% in Q4 2019, tripled to 58% in Q2 2020 and increased to 72% in Q4 2020; and in DRC, paediatric coverage increased from 16% in Q4 2019, quadrupled to 83% in Q2 2020 and increased further to 89% in Q4 2020 [16, 20]. In Mozambique, where COVID‐19 adaptations specifically addressed eligibility for children over 2 years of age, MMD coverage among clients < 15 years was 9% in Q4 2019, increased four‐fold to 38% in Q2 2020 before dipping slightly to 35% in Q4 2020 [26]. In Zambia, where the government issued guidance in March 2020 to provide 6MMD to stable clients over 10 years of age, 6MMD coverage started at 26% in Q4 2019, doubled to 54% in Q2 2020 and increased slightly to 55% in Q4 2020 [31].

### 3.4 Changes in treatment outcomes

PEPFAR program data indicate that as MMD eligibility criteria and enrolment expanded, virologic suppression rates remained high. Across the 21 countries, virologic suppression was 90% in Q4 2019 and Q1 2020 before MMD expansion (6.6 and 6.9 million clients, respectively) and steadily increased from 91% in Q2 2020 (7.0 million clients), to 92% in Q3 2020 (7.4 million clients) and to 93% in Q4 2020 (8.1 million clients). Among clients < 15 years old, virologic suppression was 71% in Q4 2019 (286,000 clients) and steadily increased to 80% in Q4 2020 (326,000 clients). Consistently increasing rates of viral suppression were maintained across nearly all 21 countries.

### 3.5 Limitations

This study has a number of limitations. First, PEPFAR began collecting MMD data in Q4 2019 giving us only two quarters of data (Q4 2019; Q1 2020) to establish a baseline prior to the COVID‐19 response; and several countries began implementing adaptations to MMD policy in March of Q1 2020. Second, MMD was a PEPFAR priority prior to COVID‐19 and uptake was increasing globally. Third, the COVID‐19 MMD policy desk review and analysis may not have captured all country‐specific policy changes; the absence of a documented country policy adaptation does not mean one did not exist, only that we could not find one. Fourth, the viral load results are promising but will need to be monitored over a longer time in order to assess impact. Lastly, we recognize that MMD is just one enabler of differentiated service delivery for HIV treatment in a suite of services and is part of an overall strategy to separate clinical care from drug distribution.

## 4 CONCLUSIONS

The COVID‐19 adaptations to MMD policy created an enabling environment for accelerating MMD uptake and extending dispensing intervals, particularly among clients < 15 years of age. Increasing access to MMD for children, pregnant and/or breastfeeding women, clients not meeting the criteria for “stable on ART” and new ART initiators supports treatment continuity while mitigating potential exposure to COVID‐19 at health facilities. Early evidence suggests that this model is beneficial in public health emergencies and during disruptions to the healthcare system. Outside emergency contexts, expanded MMD eligibility extends client‐centred care to previously excluded populations promoting improved client satisfaction, virologic suppression and treatment continuity. With millions of new clients receiving the benefits of MMD, the global HIV community can move beyond limiting these policy changes to temporary protective measures during a global pandemic and consider institutionalizing them to become the new standard of care, even as COVID‐19 subsides.

## COMPETING INTERESTS

There are no competing interests.

## AUTHORS' CONTRIBUTIONS

The initial concept for this commentary was conceived by all authors (LEB, GKS, CG, PA, MD and JRC). LEB, GKS and CG contributed to the initial outline. LEB, GKS and CG contributed to the initial manuscript content. MD conducted the statistical analysis in R. LEB, GKS, CG, MD and PA contributed to the revisions. All authors (LEB, GKS, CG, PA, MD and JRC) approved the final commentary.

## FUNDING

This article was made possible by the support of the American people through the U.S. President's Emergency Plan for AIDS Relief (PEPFAR).

## DISCLAIMER

The views expressed in this article are those of the authors and do not necessarily reflect the view of the U.S. President's Emergency Plan for AIDS Relief, the U.S. Agency for International Development, the U.S. Office of the Global AIDS Coordinator and Health Diplomacy, the United States Army, the Department of Defense, Henry M. Jackson Foundation for the Advancement of Military Medicine or the U.S. Government.
